# Characterisation of the biochemical and cellular roles of native and pathogenic amelogenesis imperfecta mutants of FAM83H

**DOI:** 10.1016/j.cellsig.2020.109632

**Published:** 2020-08

**Authors:** Theresa Tachie-Menson, Ana Gázquez-Gutiérrez, Luke J. Fulcher, Thomas J. Macartney, Nicola T. Wood, Joby Varghese, Robert Gourlay, Renata F. Soares, Gopal P. Sapkota

**Affiliations:** a Medical Research Council Protein Phosphorylation and Ubiquitylation Unit, University of Dundee, Dundee, United Kingdom; b University of Seville, Av. Sanchez Pizjuan, s/n, 41009, Seville, Spain

**Keywords:** BAG3, CK1, Iporin, NCK, TurboID, ADHCAI, autosomal dominant hypocalcified amelogenesis imperfecta, aGFP_6M_, anti-GFP nanobody with six destabilising mutations [45], AI, amelogenesis imperfecta, AP4, Adapter Protein Complex 4, BAG3, BAG family molecular chaperone regulator 3, BMP, bone morphogenetic protein, CD2AP, CD2 associated protein, CK1, Protein kinase CK1, DUF1669, domain of unknown function 1669, EGFP, enhanced green fluorescent protein, FAM83, FAMily with sequence similarity 83, HA, homology arm, IB, immunoblot, IP, immunoprecipitate, Iporin, Interacting protein of Rab1, LC-MS/MS, Liquid Chromatography tandem mass spectrometry, PACK1, Protein Anchor of CK1, PAWS1, Protein associated with SMAD1, RUSC2, RUN and SH3 domain containing protein 2, NESCA, New molecule containing SH3 at the carboxy-terminus, SMAD1, Mothers against decapentaplegic homolog 1, UNC45A, Uncoordinated 45 homolog A

## Abstract

The majority of mutations identified in patients with amelogenesis imperfecta have been mapped to *FAM83H*. As *FAM83H* expression is not limited to the enamel, how FAM83H contributes to amelogenesis is still largely unknown. We previously reported that members of the FAM83 family of proteins interact with and regulate the subcellular distribution of the promiscuous serine-threonine protein kinase CK1 family, through their shared N-terminal DUF1669 domains. FAM83H co-localises with CK1 isoforms to speckle-like structures in both the cytoplasm and nucleus. In this report, we show FAM83H, unlike other FAM83 proteins, interacts and colocalises with NCK1/2 tyrosine kinase adaptor proteins. This interaction is mediated by proline-rich motifs within the C-terminus of FAM83H, specifically interacting with the second and third SH3 domains of NCK1/2. Moreover, FAM83H pathogenic AI mutant proteins, which trigger C-terminal truncations of FAM83H, retain their interactions with CK1 isoforms but lose interaction with NCK1/2. These AI mutant FAM83H proteins acquire a nuclear localisation, and recruit CK1 isoforms to the nucleus where CK1 retains its kinase activity. As understanding the constituents of the FAM83H-localised speckles may hold the key to unravelling potential substrates of FAM83H-associated CK1 substrates, we employed a TurboID-based proximity labelling approach and uncovered several proteins including Iporin and BAG3 as potential constituents of the speckles.

## Highlights

• FAM83H interacts with CK1 isoforms through its DUF1669 domain and NCK1/2 through its C-terminus.
• *FAM83H* AI mutants retain interaction with CK1 but lose interaction with NCK1/2.
• Unlike wild type FAM83H, the AI mutants and associated CK1 isoforms localise mainly to the nucleus.
• CK1 associated with *FAM83H* AI mutants retains catalytic activity.

## 1 Introduction

FAM83H is a member of the FAMily with sequence similarity 83 (FAM83) protein family, which comprises eight family members designated FAM83A-H. FAM83 proteins are related by a common N-terminal domain of unknown function called DUF1669. The function of this protein family is mostly uncharacterised, however we have recently identified that FAM83 proteins act as scaffolding proteins for protein kinase CK1 isoforms α, δ and ε, where FAM83 proteins serve to direct CK1 isoforms to distinct subcellular compartments. The FAM83:CK1 interaction is mediated through the DUF1669 domain, which we renamed Protein Anchor of CK1 (PACK1) [1,2].

Outside of the PACK1 domain, the FAM83 proteins are unique and share no sequence similarity or homology. Consistent with this, we have found that different FAM83 members have unique interacting partners and cellular roles [[2], [3], [4], [5], [6]]. By employing a proteomics approach, we identified FAM83G, also known as Protein Associated with SMAD1 (PAWS1), as a novel interactor of SMAD1 and a novel component of the BMP signaling pathway [3]. Moreover, through its association with CK1α, PAWS1 can activate the Wnt signaling pathway [5] and mutations in PAWS1 that are implicated in palmoplantar keratoderma, lose interaction with CK1α and consequently have attenuated Wnt signaling [7]. Interestingly, we found that the interaction between FAM83D and CK1α was essential for proper positioning of mitotic spindles, and in the process uncovered CK1α as a novel mitotic kinase with important functions in cell division [6].

**Fig. 1:**
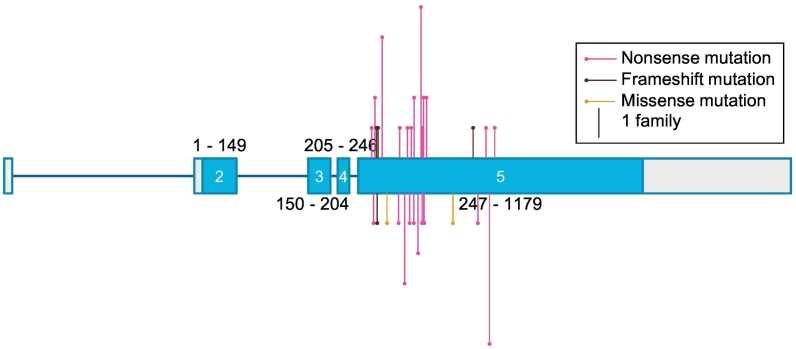
FAM83H AI mutations. Schematic diagram representing the genomic location of the mutations identified in the *FAM83H* gene implicated in amelogenesis imperfecta. Exons are represented by boxes, where coding regions are shaded in blue, and non-coding regions shaded in grey. Numbers correspond to the number of the nucleotide base pair. Introns are represented by straight blue lines. The length of vertical lines indicates the number of families reported with the mutation.

FAM83H has also been reported to be overexpressed in several types of cancers, including colorectal cancer [8,9], hepatocellular carcinoma [10] and prostate cancer [11]. Nevertheless, FAM83H is the only FAM83 member implicated in amelogenesis, as mutations in *FAM83H* have been identified in patients with autosomal dominant hypocalcified amelogenesis imperfecta (ADHCAI) [[12], [13], [14], [15], [16], [17], [18], [19], [20], [21], [22], [23], [24], [25], [26], [27], [28], [29], [30], [31]]. Amelogenesis imperfecta (AI) refers to genetic conditions in which enamel formation is compromised. This affects the appearance and structure of the enamel of primary and secondary dentition and consequently has detrimental effects on the psychosocial health of those impacted. The hypocalcified phenotype is thought to be the most severe form of AI in which enamel has normal thickness, but is soft, discoloured and wears away shortly after eruption. Prior to 2008, causative genetic mutations for ADHCAI had not been identified in genes that had previously been implicated in AI or known to be involved in amelogenesis. Thus, novel candidate genes whose mutations could explain the pathogenesis of AI were sought after. The putative disease locus was narrowed down to a 2.1 Mb region composed of 91 genes on chromosome *8q 24.3* between *FAM135B* and the telomere [32]. Through sequencing 42 genes in that 2.1 Mb region, two nonsense mutations were mapped to the terminal exon of FAM83H, *Exon 5* [12]. At present, over 20 mutations in *FAM83H*, all mapped to *Exon 5*, have been reported (Fig. 1). With the exception of two missense mutations [16,17], all other mutations are predicted to encode a premature stop codon and consequently encode a truncated FAM83H protein. All mutations affect the protein outside the DUF1669/PACK1 domain between and including residues S287 and E694 [26].

FAM83H is not solely expressed during amelogenesis and is thought to be expressed ubiquitously [12,23]. It has not previously been implicated in amelogenesis and therefore the significance of FAM83H mutations in amelogenesis imperfecta and why there is an absence of non-dental phenotypes in patients with these mutations remains a mystery. As the C-terminus of FAM83H is lost in these FAM83H truncation mutants, it is predicted that the C-terminus of FAM83H is important for the correct calcification of enamel [13], however the exact roles of FAM83H in amelogenesis are unknown. FAM83H is not expected to be secreted into the enamel matrix as it lacks a secretory signal peptide and is therefore expected to have intracellular roles in ameloblasts. However, whether FAM83H mainly functions during the pre-secretory, secretory or maturation stage of amelogenesis remains unclear [23,33].

In this study, we sought to characterise the role of the FAM83H protein and how the AI mutants modulate FAM83H function. We have employed a combination of proteomic, biochemical and cellular approaches to dissect the interactors and subcellular distribution of FAM83H, and the AI mutants and assess their impact on CK1 kinase activity.

## 2 Materials and methods

### 2.1 Plasmids

Recombinant DNA procedures were performed using standard protocols as described previously [34]. Constructs for transient transfection were subcloned into pcDNA5-FRT/TO vectors and constructs for retroviral transfection were subcloned into a pBABE vector with either EGFP, FLAG or an mCherry tag at the N or C-terminus as indicated. All constructs are available to request from the Medical Research Council (MRC) – Phosphorylation and Ubiquitylation Unit (PPU) Reagents webpage (http://mrcppureagents.dundee.ac.uk) and the unique identifier (DU) numbers indicated below provide direct links to the cloning strategy and sequence information. The following constructs were generated:

pcDNA5-FRT/TO GFP (DU 41455), pcDNA5-FRT/TO FLAG empty (DU 41457), pCMV GAG/POL (Clontech), pCMV VSV-G (Clontech) pcDNA5-FRT/TO CK1α-mCherry (DU 28469), pcDNA5-FRT/TO mCherry-CK1α (DU 28407), pcDNA5-FRT/TO GFP-FAM83H (DU 44239), pcDNA5-FRT/TO GFP-FAM83H^D236A^ (DU 28428), pcDNA5-FRT/TO GFP-FAM83H^F270A^ (DU 28487), pcDNA5-FRT/TO FLAG-FAM83H^M1-E50^ (DU 29071), pcDNA5-FRT/TO FLAG-FAM83H^P301-F350^ (DU 29070), pcDNA5-FRT/TO FLAG-FAM83H^I898-A948^ (DU 29068), pcDNA5-FRT/TO FLAG-FAM83H^A948-P999^ (DU 29069), pcDNA5-FRT/TO FLAG-FAM83H (DU 28811), pcDNA5-FRT/TO FLAG-FAM83H^D236A^ (DU 28893), pcDNA5-FRT/TO FLAG-FAM83H^F247A^ (DU 28890), pcDNA5-FRT/TO FLAG-FAM83H^F251A^ (DU 28892), pcDNA5-FRT/TO FLAG-FAM83H^F270A^ (DU 28827), pcDNA5-FRT/TO FLAG-FAM83H^F274A^ (DU 28822), pcDNA5-FRT/TO FLAG-FAM83H^F278A^ (DU 28820), pcDNA5-FRT/TO FLAG-FAM83H^F350A^ (DU 28889), pcDNA5-FRT/TO FLAG-FAM83H^F354A^ (DU 28821), pcDNA5-FRT/TO FLAG-FAM83H^A31E^ (DU 29553), pcDNA5-FRT/TO FLAG-FAM83H^S287X^ (DU 28824), pcDNA5-FRT/TO FLAG-FAM83H^S287X, D236A^ (DU 64222), pcDNA5-FRT/TO FLAG-FAM83H^Q452X^ (DU 28826), pcDNA5-FRT/TO FLAG-FAM83H^E694X^ (DU 28825), pcDNA5-FRT/TO FLAG-FAM83H^S342T^ (DU 29598), pcDNA5-FRT/TO FLAG-FAM83H^G557C^ (DU 29599), pcDNA5-FRT/TO mCherry-NCK1 (DU 28583), pcDNA5-FRT/TO NCK1-mCherry (DU 28689), pcDNA5-FRT/TO mCherry-NCK2 (DU 28691), pcDNA5-FRT/TO FLAG-NCK1 (DU 28906), pcDNA5-FRT/TO FLAG-NCK1^R308M^ (DU 28921), pcDNA5-FRT/TO FLAG-NCK1^W39K^ (DU 28922), pcDNA5-FRT/TO FLAG-NCK1^W39K, W143K^ (DU 28927), pcDNA5-FRT/TO FLAG-NCK1^W39K, W143K, W229K^ (DU 28947), pcDNA5-FRT/TO FLAG-NCK1^W39K, W229K^ (DU 28929), pcDNA5-FRT/TO FLAG-NCK1^W143K^ (DU 28923), pcDNA5-FRT/TO FLAG-NCK1^W144K, W229K^ (DU 28954), pcDNA5-FRT/TO FLAG-NCK1^W229K^ (DU 28925), pcDNA5-FRT/TO FLAG-NCK2 (DU 28907), pcDNA5-FRT/TO FLAG-NCK2^R311M^ (DU 28935), pcDNA5-FRT/TO FLAG-NCK2^W39K^ (DU 28936), pcDNA5-FRT/TO FLAG-NCK2^W39K, W149K^ (DU 28939), pcDNA5-FRT/TO FLAG-NCK2^W39K, W149K, W235K^ (DU 28948), pcDNA5-FRT/TO FLAG-NCK2^W39K, W235K^ (DU 28955), pcDNA5-FRT/TO FLAG-NCK2^W149K^ (DU 28937), pcDNA5-FRT/TO FLAG-NCK2^W39K, W149K^ (DU 28940), pcDNA5-FRT/TO FLAG-NCK2^W235K^ (DU 28938), pBABED puro FLAG-aGFP_6M_ (DU 57701), pBABED puro FLAG-TurboID (DU 29701), pBABED puro FLAG-TurboID-aGFP_6M_ (DU 29702). pBABED puro (DU 33932). Constructs used in CRISPR/Cas9 gene editing pBABED-Puro-sgRNA1 FAM83H KO sense guide RNA (gRNA; DU52010) and pX335-Cas9-D10A-sgRNA2 FAM83H KO antisense gRNA (DU52026) for knockout of FAM83H; pBABED-Puro-sgRNA1 FAM83H C-terminal KI sense gRNA (DU 54452), pX335-Cas9-D10A-sgRNA2 FAM83H C-terminal KI antisense gRNA (DU54457), pMK-RQ FAM83H GFP donor (DU 54697) for endogenous C-terminal knockin of GFP. pBABED-Puro-sgRNA1 *CSNK1A1* N-terminal KI sense gRNA (DU 57522), pX335-Cas9-D10A-sgRNA2 *CSNK1A1* N-terminal KI antisense gRNA (DU 57527), pMK-RQ *CSNK1A1* mCherry donor (DU 57578) for endogenous N-terminal knockin of mCherry to *CSNK1A1*. pBABED-Puro-sgRNA1 *CSNK1D* N-terminal KI sense gRNA (DU 57523), pX335-Cas9-D10A-sgRNA2 *CSNK1D* N-terminal KI antisense gRNA (DU 57528), pMK-RQ *CSNK1D* mCherry donor (DU 57702) for endogenous N-terminal knockin of mCherry to *CSNK1D*. pBABED-Puro-sgRNA1 *CSNK1E* N-terminal KI sense gRNA (DU 54377), pX335-Cas9-D10A-sgRNA2 *CSNK1D* N-terminal KI antisense gRNA (DU 54383), pMK-RQ *CSNK1E* mCherry donor (DU 57623) for endogenous N-terminal knockin of mCherry to *CSNK1E*. Sequences of constructs were verified by the DNA Sequencing Service, University of Dundee (www.dnaseq.co.uk).

### 2.2 Antibodies

Primary antibodies used in Western blotting were diluted in either 5% milk/TBS-T (50 mM Tris-HCl (pH 7.5), 150 mM NaCl, 0.1% (v/v) Tween 20) or 5% BSA/TBS-T and are as follows: anti-alpha-tubulin (MA1–80189, Thermo Scientific, rat monoclonal, 1:2000, BSA), anti-CK1α (A301-991A, Bethyl, rabbit polyclonal 1:1000, milk), anti-CK1α (SA527, MRC-PPU Reagents, sheep polyclonal, 1:1000, milk) anti-CK1δ (SA609, MRC-PPU Reagents, sheep polyclonal, 1:1000, milk), anti-CK1ε (HPA026288, Sigma, rabbit polyclonal, 1:1000, milk), anti-CK1ε (SA610, MRC-PPU Reagents, sheep polyclonal, 1:1000, milk) anti-FAM83H (SA273, MRC-PPU Reagents, sheep polyclonal, 1:1000, milk), anti-FLAG M2-Peroxidase (A8592, Sigma, mouse monoclonal, 1:2000, milk) anti-GAPDH (2118, Cell Signaling Technology, rabbit monoclonal, 1:2000, BSA), anti-GFP (S268B, MRC-PPU Reagents, sheep polyclonal, 1:2000, milk), anti-mCherry (ab183628 Abcam, rabbit polyclonal, 1:1000, milk), anti-NCK1 (2319, Cell Signaling Technology, rabbit monoclonal, 1:500, milk) and Streptavidin-HRP (89880D, Pierce, 1:2000, 5% BSA/TBS).

Secondary antibodies used in Western blotting were diluted in 5% milk/TBS-T and are as follows: horse anti-mouse IgG HRP (7076, Cell Signaling Technology, 1:5000), goat anti-rabbit IgG HRP (7074, Cell Signaling Technology 1:5000), rabbit anti-sheep IgG HRP (31480, Thermo Fisher Scientific, 1:5000), goat anti-rat IgG (H+L) IRDye® 800 CW (P/N 925-68029, Licor, 1:10000), StarBright Blue 700 goat anti-rabbit IgG (12004161, Bio-Rad, 1:10000).

Antibodies used in immunofluorescence were diluted in 3% BSA/PBS and are as follows: anti-FLAG (14,793, Cell Signaling Technology, rabbit monoclonal, 1:400), Alexa Fluor 488 donkey anti rabbit IgG (H + L) (A21206, Cell Signaling Technology, 1:300), Alexa Fluor 488 donkey anti sheep IgG (H + L) (A11015, Cell Signaling Technology, 1:300), Alexa Fluor 488 goat anti rabbit IgG (H + L) (A11034, Cell Signaling Technology, 1:300), Alexa Fluor 594 donkey anti goat IgG (H + L) (A11058, Cell Signaling Technology, 1:300), Alexa Fluor 594 donkey anti-sheep IgG (H + L) (A11016, Cell Signaling Technology, 1:300), Streptavidin, Alexa Fluor 594 conjugate (S32356, Life Technologies, 1:300).

### 2.3 Cell culture

U2OS osteosarcoma (HTB-96, ATCC), HEK-293 human embryonic kidney (CRL-1573, ATCC) and A549 human pulmonary adenocarcinoma (CCL-185, ATCC) cells were grown in Dulbecco's modified Eagle's medium (DMEM: 11960–085, GIBCO) supplemented with 10% (v/v) foetal bovine serum (FBS) (F7524, Sigma), 2 mM l-glutamine (25030024, GIBCO), 100 U/mL penicillin-streptomycin (15140122, GIBCO). Cells were incubated at 37 °C, 5% CO_2_ in a humidified atmosphere.

For transient transfections, 20 μl of polyethylenimine (PEI, 1 mg/ml) (24765, Polysciences Inc) and 2 μg of plasmid DNA was added to 1 mL of Opti-Mem (31985–062, GIBCO). This mixture was then gently vortexed for 20 s then incubated at room temperature for 20 min. After incubation, the transfection mixture was added drop by drop to the media of cells in a 10 cm dish.

To induce the expression of FAM83-GFP or GFP-FAM83 proteins in U2OS or HEK-293 Flp-In T-Rex cells respectively, cells were incubated in 20 ng/mL doxycycline for 24 h.

### 2.4 Generation of stable Flp-In T-Rex cell lines

U2OS or HEK-293 cell lines stably expressing FAM83 proteins with a C or N-terminal GFP tag, respectively, were generated as described previously [1].

### 2.5 Generation of U2OS FAM83H^−/−^ using CRISPR/Cas9

Generation of U2OS *FAM83H*^*−/−*^ has been previously described [1]. To summarise, CRISPR/Cas9 gene editing technology was used to knockout *FAM83H* in U2OS cells. U2OS cells at 60% confluency grown in a 10 cm dish were transfected with 1 μg of two separate plasmid vectors encoding antisense and sense guide RNAs (pBABED-Puro-sgRNA1 FAM83H KO sense guide RNA (gRNA; DU52010) and pX335-Cas9-D10A-sgRNA2 FAM83H KO antisense gRNA (DU52026)) that target the first coding exon of *FAM83H*, Exon 2. After 16 h, the media of the cells were changed and cells were selected in 2 μg/mL puromycin (P9620, Sigma) for two days. The transfection was repeated and 16 h after this transfection the cell media was replaced with fresh media. Cells were isolated by single cell sorting and were plated into separate wells of two 96-well plates precoated with 1% (w/v) gelatine (G7765, Sigma). Cell clones that survived and proliferated were expanded and knockout was confirmed by Western blotting and DNA sequencing of the genomic region targeted by CRISPR/Cas9.

### 2.6 Generation of A549 FAM83H^GFP/GFP^ and U2OS CK1^mCherry/mCherry^ using CRISPR/Cas9

A549 cells were transfected with 1 μg of each plasmid vector encoding antisense and sense guide RNAs for *FAM83H* (pBABED-Puro-sgRNA1 *FAM83H* C-terminal KI sense (DU 54452), pX335-Cas9-D10A-sgRNA2 *FAM83H* C-terminal KI antisense (DU54457)), which target the region proximal to the stop codon of *FAM83H*, and 3 μg of a donor plasmid (pMK-RQ *FAM83H* GFP donor (DU 54697)), which contains the gene encoding enhanced GFP (EGFP) flanked by 5′ and 3′ homology arms. 16 h after transfection, cells were selected in 2 μg/mL puromycin (Sigma, P9620) for 48 h. The transfection was repeated. 16 h after this transfection, the cell media was replaced with fresh media. Fluorescence activated cell sorting was employed to isolate GFP-positive cells. Single GFP-positive cell clones were plated into single wells of two 96-well plates precoated with 1% (w/v) gelatine (G7765, Sigma). Surviving cell clones were expanded and endogenous knockin of GFP to the C-terminus of *FAM83H* was verified by Western blotting and genomic sequencing of the targeted locus.

For knockin in the mCherry tag to endogenous CK1 isoforms, the same protocol was performed using U2OS cells except with the following constructs: pBABED-Puro-sgRNA1 *CSNK1* N-terminal KI sense gRNAs (DU 57522, DU 57523 or DU 54377), pX335-Cas9-D10A-sgRNA2 *CSNK1* N-terminal KI antisense gRNA (DU 57527, DU 57528 or DU 54383), which target the 3′ end of the 5′UTR and the start of exon 1 *CSNK1A1*, *CSNK1D* or *CSNK1E*, and 3 μg of a donor plasmid (pMK-RQ CSNK1 mCherry donor (DU 57578, DU 57702 or DU 57623), which contains the gene encoding mCherry and a 3′ 5× poly-glycine linker.

### 2.7 Generation of stable cell lines by retroviral transduction

6 μg of retroviral pBABE vectors, 3.2 μg pCMV5-GAG/POL, 2.8 μg pCMV5-VSV-G and 24 μl PEI (1 mg/ml) were added to 1 mL of Opti-Mem (31985–062, GIBCO). The mixture was vortexed gently then incubated at room temperature for 20 min. The transfection mixture was added drop by drop to HEK-293-FT cells at around 70% confluency growing within a 10 cm diameter cell culture dish. 24 h after transfection, the cell media was changed. 24 h later, the media containing retroviruses was collected and passed through 0.22 μm sterile filters. The target cell lines, at 60% confluency, were then retrovirally transduced by incubating cells with viral media, at the appropriate viral titre, in the presence of 8 μg/mL hexadimethrine bromide (also known as polybrene, H9268, Sigma) for 24 h. The cell media was then replaced with fresh media containing 2.5 μg/mL puromycin (P9620, Sigma) to select cells that had been successfully transduced and had incorporated the constructs. Cells were selected with puromycin for 48 h and then incubated in fresh culture media.

### 2.8 Cell lysis

Generally, cells were washed twice in ice-cold PBS then scraped in lysis buffer (50 mM Tris-HCl (pH 7.4), 270 mM sucrose, 150 mM NaCl, 1 mM EDTA (pH 8.0), 1 mM EGTA (pH 8.0), 1 mM sodium orthovanadate, 10 mM sodium β-glycerophosphate, 50 mM sodium fluoride, 5 mM sodium pyrophosphate, and 1% (v/v) Nonidet P40 (492016, Merck) supplemented with 1× EDTA-free protease inhibitor cocktail (11873580001, Merck). Lysates were incubated on ice for 10 min, then clarified by centrifugation at 17,000*g* for 10 min at 4 °C. The supernatant (soluble cell extract) was collected in a fresh Eppendorf and either snap frozen in liquid nitrogen and stored at −80 °C for later processing or processed immediately after the protein concentration was measured in a 96-well format using the Bradford protein assay reagent (23236, Pierce).

TurboID experiments were adapted from the method described by Branon et al. [35]. Briefly, Cells were incubated with biotin (B4501, Sigma Aldrich) for 10 min at 37 °C then washed five times in ice cold PBS. Cells were then scraped in PBS, pelleted by centrifugation at 300*g* for 3 min at 4 °C, lysed in RIPA lysis buffer supplemented with 1× EDTA-free protease inhibitor cocktail (11873580001, Merck), then clarified as described above.

### 2.9 Immunoprecipitation

For immunoprecipitation, cell extracts (300–1000 μg of protein) were incubated with 10 μL of GFP-trap A beads (gta-10, Chromotek) or M2 anti-FLAG agarose gel (A2220, Sigma) for 1 h at 4 °C with rotation. Beads were washed three times in lysis buffer or once in lysis buffer supplemented to 250 mM NaCl, then twice in lysis buffer for samples containing two or more epitope tags. Proteins were then eluted from beads by boiling beads in 1× Laemmli sample buffer containing 5% (v/v) 2-mercaptoethanol (5× Laemmli sample buffer was made as follows: 1 M Tris-HCl pH 6.8, 10% (w/v) SDS, 20% (v/v) glycerol, 0.5% (w/v) bromophenol blue, 25% (v/v) 2-mercaptoethanol, ultrapure H_2_O) at 95 °C for 5 min.

For the affinity purification pertaining to the TurboID mass spectrometry experiments, cleared cell extracts (4 mg protein) were incubated with 70 μL of streptavidin beads (17-5113-01, GE healthcare) for 16 h with rotation at 4 °C. The beads were washed twice in RIPA lysis buffer, once with 1 M KCl, once with 0.1 M Na_2_CO_3_, once with 2 M urea in 10 mM Tris-HCl pH 8.0 then twice with RIPA lysis buffer. Proteins were eluted in 70 μL 1× LDS sample buffer (NP0007, Invitrogen) containing 5 mM DTT supplemented with an additional 20 mM DTT and 2 mM biotin for 10 min at 95 °C.

### 2.10 SDS-PAGE and Western blotting

Reduced cell extracts (10 μg to 20 μg protein) or immunoprecipitates were resolved on 10% SDS-PAGE gels or 4–12% NuPAGE bis-tris gradient gels (Invitrogen) by electrophoresis. Proteins were transferred onto polyvinylidene fluoride (PVDF) membranes (10344661, Millipore), that had been pre-activated in 100% (v/v) methanol for 1 min. Membranes were blocked with 5% (w/v) non-fat milk powder (Marvel) in TBS-T, then incubated with primary antibody diluted in either 5% (w/v) milk in TBS-T or 5% (w/v) bovine serum albumin (BSA) in TBS-T for either 1 h at room temperature or 24 h at 4 °C with shaking. Membranes were washed 3 times for 10 min in TBS-T then incubated in secondary antibody for 1 h at room temperature followed by washing in TBS-T 3 times for 10 min each. Proteins were then detected by incubation with enhanced chemiluminescence reagent (WBKLS0500, Millipore) and visualised using either a Chemidoc MP system (17001402, Bio-Rad), medical-grade X-ray films (KAX1824, ScotRad), Hyperfilm (28906837, GE Healthcare) or using the Odyssey Imager (LI-COR).

### 2.11 Fluorescence microscopy

Cells were seeded onto sterile 16-mm diameter glass circle coverslips then transfected with 250 ng of the relevant plasmid DNA. 24 h after transfection, cells were washed twice in PBS then fixed in 4% (*w*/*v*) formaldehyde in PBS for 20 min at room temperature. Cells were then washed twice in PBS then permeabilised with 0.2% (v/v) Nonidet P-40 in PBS for 4 min. Cells were washed twice in PBS then blocked in blocking solution (3% (w/v) BSA in PBS) for 30 min. Cells were incubated in primary antibody diluted in blocking solution for 1 h at room temperature. Cells were then washed 3 times in PBS for 5 min per wash, then incubated in secondary antibody and 4′,6-diamidino-2-phenylindole (DAPI, Sigma Aldrich, D9542, 1 μg/mL) diluted in blocking solution for 1 h at room temperature. Cells were then washed 3 times in PBS for 5 min per wash, then washed in ultrapure water once. Coverslips were dried cell side up, then mounted using VECTASHIELD anti-fade mounting medium (H-1000, Vector Laboratories). Coverslips were sealed with Covergrip coverslip sealant (89411-108, VWR) and left to dry. Cells were visualised using a 60× lens (1- UB932, Olympus, 1.4 Lens NA) on DeltaVision Widefield Deconvolution Microscopy (Applied Precision/GE Healthcare). Images were deconvolved using softWoRx software (GE Healthcare) and processed using OMERO [36].

### 2.12 Mass spectrometry

U2OS or HEK-293 Flp-In T-Rex cells were induced to express GFP-tagged FAM83 proteins, then lysates were processed for mass spectrometry as previously described [1]. For mass spectrometry analysis of endogenous FAM83H protein-protein interactions, A549 *FAM83H*^*GFP/GFP*^ or A549 wild type cells were lysed and clarified as described above, then passed through Costar Spin-X columns (8161, Corning). Soluble cell extracts (6 mg protein) were incubated with 12 μL of GFP-Trap A beads (gta-10, Chromotek) for 16 h as described above. Proteins were eluted and resolved using SDS-PAGE on a 4 to 12% NuPAGE bis-tris gradient gel (Invitrogen). The gel was stained using InstantBlue (ISB1L, Expedeon), then destained in ultrapure water. Gel pieces were excised, and proteins digested with trypsin. Peptides were then analysed by mass spectrometry using Liquid Chromatography-tandem Mass spectrometry (LC-MS/MS) on a Linear ion trap-orbitrap hybrid mass spectrometer (Orbitrap-VelosPro, Thermo) coupled to a U3000 RSLC HPLC (Rapid Separation/High Performance Liquid Chromatography; Thermo). See Fulcher et al. [1] for a more detailed description of the protocol.

### 2.13 Protein expression and purification

GST-PAWS1-6xHis (DU 28293, MRC-PPU reagents) protein was obtained from Dr. P. Bozatzi. The protein was first expressed in BL21 (DE3) *Escherichia. coli* as previously described [1], then affinity-purified sequentially using reduced glutathione-Sepharose and nickel-agarose columns.

### 2.14 Immunoprecipitation-in vitro kinase assay

After washing in lysis buffer three times, beads were washed once in 1 mL of 10 X kinase assay buffer (10× kinase assay buffer: 50 mM Tris-HCl (pH 7.5), 0.1 mM EGTA, 10 mM magnesium acetate, 2 mM dithiothreitol (DTT)). 10 μL of beads was resuspended in 5 μL 10× kinase assay buffer, 2 μg GST-PAWS1-6xHis (DU 28293, MRC-PPU reagents) and made up to a total volume of 25 μL. Reactions were then incubated with 0.1 mM [^γ32^P]-ATP (~500 cpm/pmol) for 30 min at 30 °C. Reactions were quenched upon addition of 1× Laemmli sample buffer containing 5% (v/v) 2-mercaptoethanol and boiling at 95 °C for 5 min. Samples were resolved on a 4–12% NuPAGE bis-tris gradient gel (Invitrogen) by SDS-PAGE. Gels were stained with InstantBlue (ISB1L, Expedon), then destained in ultrapure water then dried. Radioactivity was assessed by exposure of the gel to Hyperfilm (28906837, GE Healthcare) overnight at −80 °C.

## 3 Results

### 3.1 FAM83H interacts with NCK1/2

**Fig. 2:**
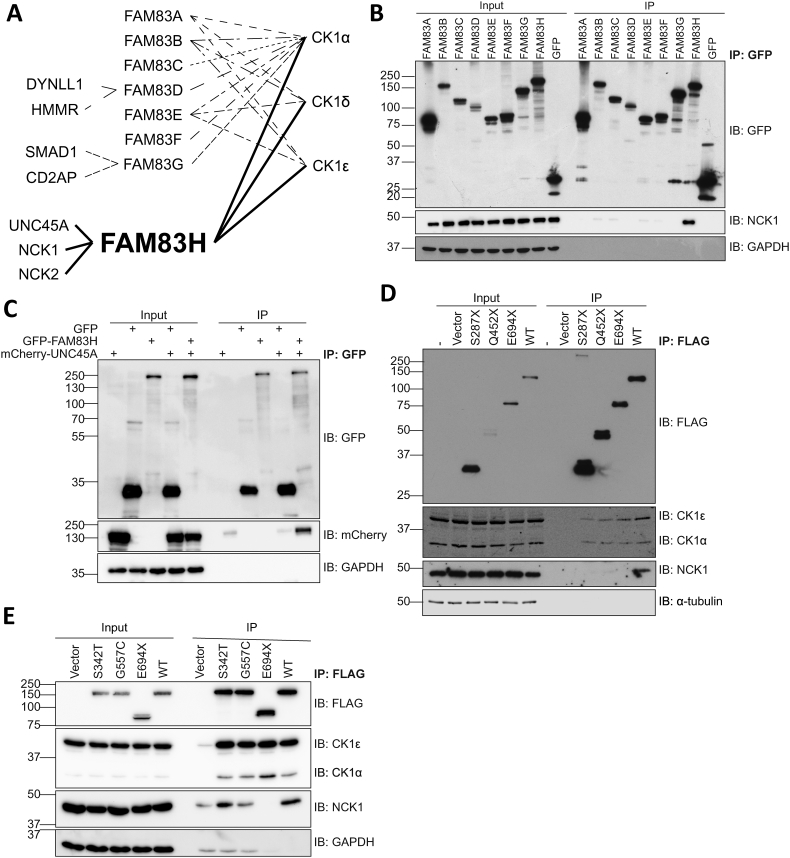
FAM83H interacts with NCK adaptor proteins. **A** Schematic diagram showing FAM83 proteins interact with CK1 isoforms and have unique interactors. **B** HEK-293 Flp-In T-REx cells that express GFP-FAM83 proteins under a doxycycline inducible promoter were treated with 20 ng/mL doxycycline for 24 h and GFP was pulled down from lysates then cell extracts (input) and pull downs (IP) were analysed by immunoblotting (IB). *n* = 3. **C** U2OS *FAM83H^-/-^* cells were transiently transfected with vectors encoding GFP, GFP-FAM83H and/or mCherry-UNC45A and lysates subjected to anti-GFP pull downs. Cell extracts (input) and pull downs (IP) were then analysed by Western blotting. *n* = 2. **D** Empty FLAG vector (vector), FLAG-FAM83H^S287X^, FLAG-FAM83H^Q452X^, FLAG-FAM83H^E694X^ and FLAG-FAM83H^WT^ were transiently transfected into U2OS *FAM83H^-/-^* cells. Untransfected U2OS *FAM83H^-/-^* cells (−) were used as an additional control. Anti-FLAG immunoprecipitations were performed on lysates and cell extracts (input) and immunoprecipitates (IP) were analysed by Western blotting. n = 3. **E** FLAG empty vector (vector), FLAG-FAM83H^S342T^, FLAG-FAM83H^G557C^, FLAG-FAM83H^E694X^ and FLAG-FAM83H^WT^ were transiently transfected into HEK-293 wild type cells. Anti-FLAG pull downs were performed on lysates then cell extracts (input) and immunoprecipitates (IP) were analysed by Western blotting. *n* = 1.

We have previously employed a proteomic approach to identify the interactors of each FAM83 member using HEK-293 and U2OS osteosarcoma Flp-In T-REx cells stably expressing each FAM83 member with an N- or C-terminal GFP tag respectively, under control of a tetracycline inducible promoter [1]. Proteins associated with anti-GFP immunoprecipitates (IPs) for each FAM83 protein were identified by LC-MS/MS (project PXD009335, PRIDE database (https://www.ebi.ac.uk/pride/) [37]). We found that all FAM83 members interact with protein kinase CK1 isoform α (CK1α), while FAM83A, B, E and H also interacted with CK1 isoforms δ and ε (Fig. 2A) [1]. We have verified the interaction between FAM83D and CK1α in mitosis and demonstrated that it is necessary for correct spindle positioning in mitosis, and also confirmed the interaction between FAM83D and DYNLL1 and HMMR [6]. Similarly, we have validated the interactions of PAWS1 with CK1α [5], CD2AP [4] and SMAD1 [3] (Fig. 2A). From the proteomics data, the standout unique interactors identified for FAM83H were protein UNC45 homolog A (UNC45A) and non-catalytic region of tyrosine kinase adaptor proteins 1 and 2 (NCK1 and NCK2) (Fig. 2A). Indeed, endogenous NCK1 was identified by immunoblotting in GFP-FAM83H IPs of cell extracts from HEK-293 Flp-In T-REx cells induced to express GFP-FAM83H under a tetracycline inducible promoter (Fig. 2B). mCherry-UNC45A similarly co-precipitated with GFP-FAM83H when transiently expressed in U2OS *FAM8H^-/-^* (generated using CRISPR/Cas9 technology [38]), but not with GFP control (Fig. 2C).

All but two AI mutations that are mapped to *FAM83H* lead to the expression of truncated FAM83H proteins (Fig. 1). We investigated the effects of these pathogenic mutations on the association with FAM83H interactors. Firstly, we generated vectors encoding truncated FAM83H proteins with an N-terminal FLAG tag that correspond to the three mutations in FAM83H identified in some patients with ADHCAI, namely FLAG-FAM83H^S287X^, FLAG-FAM83H^Q452X^ and FLAG-FAM83H^E694X^. S287X encodes the smallest truncated protein, Q452X is one of the most frequently reported AI mutations, and E694X encodes the largest truncated protein. These vectors were transiently transfected into U2OS *FAM83H^-/-^* cells and extracts subjected to anti-FLAG IPs. All three mutants retained interaction with CK1 isoforms, but lost interaction with NCK1 (Fig. 2D). Two missense mutations FAM83H^S342T^ and FAM83H^G557C^ have also been reported in the literature, although the accuracy in mapping these mutations has been questioned [39]. These missense mutations, like wild type FAM83H, interacted with CK1 isoforms and NCK (Fig. 2E).

### 3.2 The interaction between FAM83H and CK1 is not mediated by the canonical F-x-x-x-F motif

**Fig. 3:**
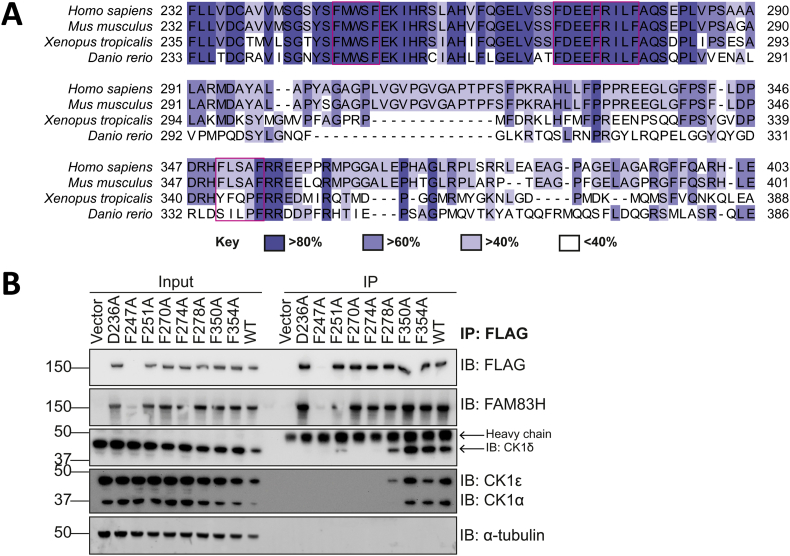
The interaction between FAM83H and CK1 is not mediated by the canonical F-x-x-x-F motif. **A** Sequence alignment of *Homo sapiens* (human), *Mus musculus* (mouse), *Xenopus tropicalis* and *Danio rerio* FAM83H proteins. Shading indicates percentage sequence identity as shown in key. F-x-x-x-F motifs are highlighted by a magenta box. Generated using ClustalO [58] in Jalview [59]. **B** U2OS *FAM83H^-/-^* cells transiently expressing FLAG empty (vector), FLAG-FAM83H^D236A^, FLAG-FAM83H^F247A^, FLAG-FAM83H^F251A^, FLAG-FAM83H^F270A^, FLAG-FAM83H^F274A^, FLAG-FAM83H^F278A^, FLAG-FAM83H^F350A^, FLAG-FAM83H^F354A^ and FLAG-FAM83H^WT^ were lysed and FLAG was immunoprecipitated from cell lysates using anti-FLAG antibodies conjugated to agarose gel. Input and IP samples were resolved by SDS-PAGE and immunoblotted (IB) with the antibodies shown. *n* = 2.

It was reported that an F-x-x-x-F motif mediates the binding of PERIOD proteins to CK1 isoforms [40], where F represents phenylalanine and x is any amino acid. FAM83H residue F274 belongs to one such conserved motif in FAM83H (F^270^-x-x-x-F^274^) and when mutated, F274A abolishes CK1 binding [9]. However, we have found that the binding between FAM83 proteins and CK1 was not solely dependent on the F-x-x-x-F motif, as mutating the conserved Phe residue of other FAM83 proteins equivalent to FAM83H^F274^ did not abolish CK1 binding [1,5], suggesting that the interaction could be mediated through a structural moiety rather than a canonical interaction motif. Human FAM83H has four F-x-x-x-F motifs and three of these motifs are highly conserved in vertebrates (Fig. 3A). We mutated all four FAM83H F-x-x-x-F motifs, in which individual Phe residues from each motif were mutated to alanine. Although we could not express FLAG-FAM83H^F247A^ successfully. Nevertheless, we found that only F270A and F274A mutations attenuated the interaction with CK1 (Fig. 3B). Moreover, D236A and A31E mutations, which do not conform to the F-x-x-x-F motif and are conserved in other FAM83 proteins, also abolished the interaction between FAM83H and CK1 isoforms, [1,7] (Fig. 3B).

### 3.3 The interaction between FAM83H and NCK is mediated by the second and third SH3 domains of NCK

**Fig. 4:**
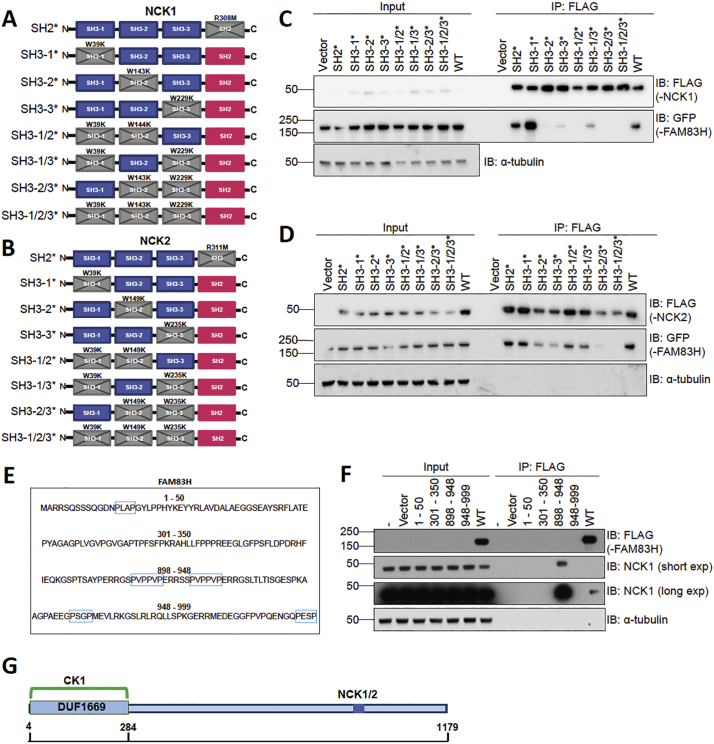
The interaction between FAM83H and NCK is mediated by the second and third SH3 domains of NCK. **A** Schematic representation of dominant negative NCK mutants generated for use in **C**. Mutated domains are indicated by crosses and are greyed out. The mutated residues are shown above the domain. W is tryptophan, K is lysine, R is arginine, and M is methionine. **B** As **A** but for NCK2 and for use in **D**. **C** A single copy of GFP-FAM83H was introduced stably downstream of a doxycycline inducible promoter in U2OS Flp-In T-REx cells. Cells were transiently transfected with FLAG empty vector (vector) FLAG-NCK2^R308M^(SH2*), FLAG-NCK2^W39K^ (SH3–1*), FLAG-NCK2^W143K^ (SH3–2*), FLAG-NCK2^W229K^ (SH3–3*), FLAG-NCK2^W39K, W144K (^SH3–1/2)*, FLAG-NCK2^W39K, W229K^ (SH3–1/3*), FLAG-NCK2^W143K, W229K^ (SH3–2/3*), or FLAG-NCK2^W39K, W143K, W229K^ (SH3–1/2/3*). Cell extracts (input) and anti-FLAG immunoprecipitates (IP) were resolved by SDS-PAGE then immunoblotted. *n* = 1. **D** As **C**, but the following constructs were used instead FLAG empty vector (vector), FLAG-NCK2^R311M^(SH2*), FLAG-NCK2^W39K^ (SH3–1*), FLAG-NCK2^W149K^ (SH3–2*), FLAG-NCK2^W235K^ (SH3–3*), FLAG-NCK2^W39K, W149K (^SH3–1/2)*, FLAG-NCK2^W39K, W235K^ (SH3–1/3*), FLAG-NCK2^W149K, W235K^ (SH3–2/3*), or FLAG-NCK2^W39K, W149K, W235K^ (SH3–1/2/3*). *n* = 2. **E** Fragments of FAM83H for **F** were generated as shown. Numbers indicate the residues of FAM83H. Blue boxes indicate proline rich motifs. **F** U2OS *FAM83H^-/-^* cells were transiently transfected with FLAG empty vector (vector), or fragments from **C** tagged with an N-terminal FLAG tag and wild type FAM83H (WT) or not transfected at all (−). Cells were lysed then subjected to anti-FLAG immunoprecipitation. Cell extracts (input) and immunoprecipitates (IP) were resolved by SDS-PAGE and immunoblotted. Exp.: exposure. n = 2. **G** Schematic diagram representing the interaction sites between FAM83H and CK1 and NCK identified by our lab.

Next, we sought to map the determinants of the FAM83H-NCK interaction. NCK1 and NCK2 comprise three N-terminal SH3 domains and one C-terminal SH2 domain; so we generated dominant negative mutants of NCK1 and NCK2 in which each domain of NCK was inactivated [41]. For NCK1, the single domain mutants were generated as follows: W39K, W143K, W229K and R308M, which inactivated the first, second and third SH3 domains (SH3-1*, SH3-2* and SH3-3*) and the SH2 domain (SH2*) respectively (Fig. 4A). Double and triple SH3 domain mutants were also generated (Fig. 4A). This was repeated for NCK2 (Fig. 4B). The NCK1/2 domain mutants alongside wild-type NCK1/2 were tagged with an N-terminal FLAG tag and transiently expressed in U2OS Flp-In T-REx cells that had been induced to express FAM83H-GFP. For NCK1, wild type, SH2* and SH3–1* robustly co-precipitated GFP-FAM83H but the SH3–2* and SH3–3* single mutants, double or SH3–1/2/3 triple mutants did not co-precipitate FAM83H-GFP (Fig. 4C). Similar results were observed for NCK2 although only SH3–2/3 double mutants did not robustly co-precipitate GFP-FAM83H (Fig. 4D).

The SH3 domains of NCK interact with proline rich motifs with a minimum consensus sequence of P-x-x-P, where P represents proline and x represents any amino acid [42,43]. Armed with the knowledge that truncating FAM83H at residue E694 disrupts the interaction of FAM83H with NCK1 (Fig. 2D), we hypothesised that NCK interacts with proline rich motifs near the C-terminus of FAM83H. There are several proline rich motifs after residue E694 of FAM83H. Therefore, 50 amino acid fragments of FAM83H comprising these proline rich motifs were generated (Fig. 4E). Two fragments were generated as negative controls: FLAG-FAM83H^M1–E50^ which comprises one proline rich motif P^14^-L^15^-A^16^-P^17^ and FLAG-FAM83H^P301-P350^, which had no proline rich motifs. U2OS *FAM83H^-/-^* cells were transiently transfected with FLAG empty vector and vectors encoding FLAG-FAM83H^I898-A948^, FLAG-FAM83H^A948-P999^ and FLAG-FAM83H^WT^ as well as FLAG-FAM83H^M1-E50^ and FLAG-FAM83H^P301-P350^ control vectors. NCK1 co-precipitated with FLAG-FAM83H^WT^ and, to a greater degree, FLAG-FAM83H^I898-A948^ but did not co-precipitate with FLAG-FAM83H^M1–E50^, FLAG-FAM83H^P301-P350^, FLAG-FAM83H^A948–P999^ and FLAG empty vector controls (Fig. 4F). Taken together, CK1 interacts with the N-terminus of FAM83H via the DUF1669/PACK1 domain and NCK interacts with the C-terminus of FAM83H (Fig. 4G).

### 3.4 Pathogenic AI FAM83H mutants co-localise with CK1 in the nucleus, but do not colocalise with NCK

**Fig. 5:**
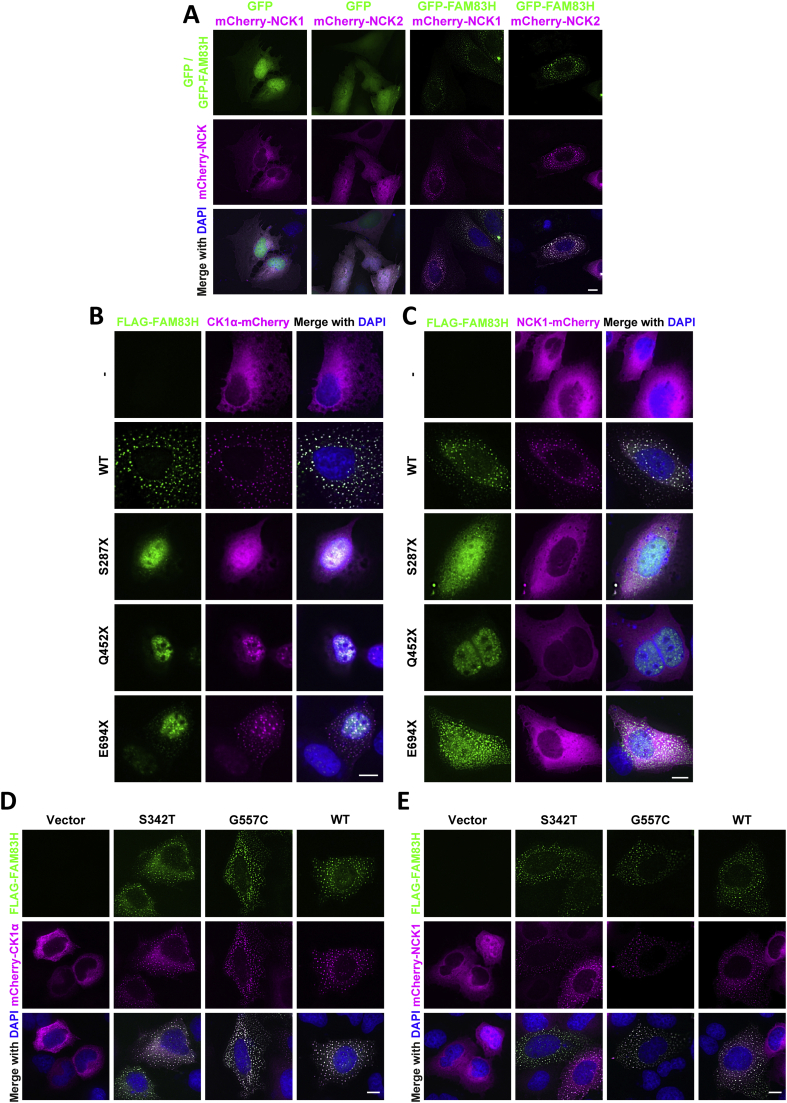
Wild type FAM83H and disease mutants co-localise with CK1, but mutants do not colocalise with NCK1. **A** U2OS *FAM83H^-/-^* cells transiently expressing GFP, GFP-FAM83H, mCherry-NCK1 or mCherry-NCK2, as indicated, were fixed and stained with 4′,6-diamidino-2-phenylindole (DAPI). Cells were imaged using the DeltaVision widefield fluorescence microscope then images were deconvolved and processed using OMERO (12). Scale bar represents 10 μm. n = 3 **B** As A but U2OS *FAM83H^-/-^* cells were transiently transfected with FLAG-FAM83H^WT^ (WT), FLAG-FAM83H^S287X^ (S287X), FLAG-FAM83H^Q452X^ (Q452X), FLAG-FAM83H^E694X^ (E694X), or no FLAG construct (−) and co-transfected with mCherry-CK1α. Scale bar represents 10 μm. n = 3 **C** As **B** but with mCherry-NCK1 instead of mCherry-CK1α. n = 2 **D** U2OS *FAM83H^-/-^* cells transiently expressing FLAG (vector), FLAG-FAM83H^S342T^, FLAG-FAM83H^G557C^, FLAG-FAM83H^WT^ and mCherry-CK1α were fixed and stained with anti-FLAG antibodies and DNA stained with DAPI. Cells were imaged using DeltaVision widefield fluorescence microscope. Scale bar represents 10 μm. n = 2 **E** As **D** but mCherry-NCK1 was used instead of mCherry-CK1α. Scale bar represents 10 μm. n = 1

It has been previously reported that FAM83H forms cytoplasmic and nuclear speckles when overexpressed and that CK1 colocalises with FAM83H at these speckles [1,9,44]. Moreover, the interaction between FAM83H and CK1 is necessary for speckle formation [1,9]. In U2OS *FAM83H^-/-^* cells, transiently expressed mCherry-NCK1 and mCherry-NCK2 colocalised with GFP-FAM83H primarily at cytoplasmic speckles, but also exhibited some non-overlapping diffuse cytosolic localisation, and did not colocalise with free, untethered GFP (Fig. 5A). Moreover, when CK1α-mCherry or NCK1-mCherry was transiently expressed in U2OS *FAM83H^-/-^* cells alongside FLAG-FAM83H wildtype or AI mutants S287X, Q452X or E694X, CK1α-mCherry colocalised with wild type and mutant FAM83H (Fig. 5B), whereas NCK1-mCherry only co-localised with WT but not mutant FAM83H (Fig. 5C). The FAM83H mutants exhibit a different localisation pattern to wild type FAM83H, as wild type FAM83H was mostly observed to localise in cytoplasmic speckles, whereas S287X and Q452X mutants mostly localise to the nucleus. However, E694X mutant, which causes a less severe AI phenotype, has both cytoplasmic and nuclear localisation (Fig. 5B, C). FAM83H AI missense mutants FAM83H^S342T^ and FAM83H^G557C^ exhibited a similar localisation pattern to wild type FAM83H where mCherry-CK1α (Fig. 5D) and mCherry-NCK1 (Fig. 5E) colocalised with these mutants.

### 3.5 CK1 associated with FAM83H^Q452X^ maintains kinase activity

**Fig. 6:**
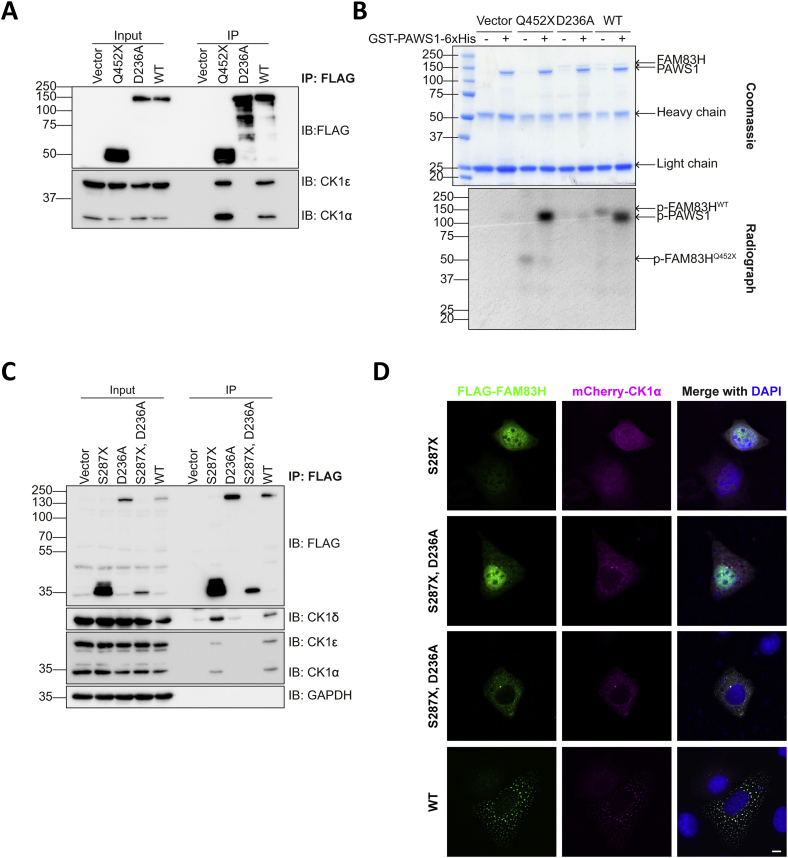
CK1 associated with FAM83H^Q452X^ maintains kinase activity. **A** FLAG empty vector (vector), FLAG-FAM83H^Q452X^, FLAG-FAM83H^D236A^ and FLAG-FAM83H^WT^ were transiently transfected into U2OS *FAM83H^-/-^* cells. Cells were then lysed and lysates subjected to anti-FLAG immunoprecipitation. Cell extract (input) and immunoprecipitates (IP) were resolved by SDS-PAGE then immunoblotted (IB) with the antibodies indicated. *n* = 2. **B** As **A** but immunoprecipitates were incubated with recombinant GST (glutathione S-transferase)-PAWS1-His in the presence of [^γ**32**^P]-ATP (adenosine 5′-triphosphate). Once the reaction was quenched, samples were resolved by SDS-PAGE. The gel was stained with InstantBlue (top) then dried and subjected to ^32^P radiography (bottom). *n* = 2. **C** U2OS *FAM83H^-/-^* cells were transiently transfected with FLAG empty vector (vector), FLAG-FAM83H^D236A^, FLAG-FAM83H^S287X^, FLAG-FAM83H^S287X, D236A^ and FLAG-FAM83H^WT^. Cells were lysed and lysates incubated with anti-FLAG beads for 16 h. Immunoprecipitates (IP) and cell extracts (input) were resolved by SDS-PAGE and proteins immunoblotted with the antibodies indicated. *n* = 2. **D** U2OS *FAM83H^-/-^* cells transiently expressing FLAG-FAM83H^S287X^, FLAG-FAM83H^S287X, D236A^, FLAG-FAM83H^WT^ and mCherry-CK1α were stained with anti-FLAG and DNA stained with DAPI. *n* = 2

Since CK1α was observed to colocalise with FAM83H disease mutants, which have an altered subcellular localisation, one could infer that these pathogenic mutants cause a mislocalisation of CK1α that may cause CK1 to phosphorylate atypical substrates within the nucleus and thereby lead to pathogenesis. Therefore, we sought to investigate whether the kinase activity of CK1 was affected by its interaction with FAM83H disease mutant Q452X. To do this, FLAG empty vector, FLAG-FAM83H^Q452X^, FLAG-FAM83H^D236A^ and FLAG-FAM83H^WT^ were transiently transfected into U2OS *FAM83H^-/-^* cells. Anti-FLAG IPs followed by Western blotting confirmed the co-precipitation of CK1α and CK1ε with Q452X and WT but not D236A nor FLAG empty vector (Fig. 6A). Under these conditions, subjecting the anti-FLAG IPs to an *in vitro* kinase assay with recombinant GST-PAWS1-His (a previously described CK1 substrate [1]) and ^32^γ-ATP, revealed that PAWS1 phosphorylation was observed with anti-FLAG IPs from both Q452X and WT extracts but not from D236A nor FLAG alone extracts (Fig. 6B).

As the speckle localisation of FAM83H is dependent on interaction with CK1 [1,9] and as CK1 maintained its kinase activity with the Q452X mutant in vitro, we sought to investigate whether this nuclear localisation of AI mutants is a result of the maintained interaction with CK1, because no obvious canonical nuclear localisation signal could be identified in any of these truncation mutants. The D236A point mutation was introduced to the S287X mutant. To validate this mutant does not interact with CK1, we performed co-IP experiments. FLAG empty vector, FLAG-FAM83H^D236A^, FLAG-FAM83H^S287X^, FLAG-FAM83H^S287X, D236A^ and FLAG-FAM83H^WT^ were transiently transfected into U2OS *FAM83H^-/-^* cells. As expected, CK1α and CK1ε did not co-IP with FLAG control, FLAG-FAM83H^D236A^ nor FLAG-FAM83H^S287X, D236A^, but did co-IP with FLAG-FAM83H^S287X^ and FLAG-FAM83H^WT^ (Fig. 6C). The co-localisation of mCherry-CK1α and FLAG-FAM83H^S287X^ was compared with that of FLAG-FAM83H^S287X, D236A^. FLAG-FAM83H^S287X^ had a predominantly nuclear localisation with overlapping mCherry-CK1α localisation (Fig. 6D). Interestingly, FLAG-FAM83H^S287X, D236A^ could be observed to have two different localisation patterns; some cells had a purely diffused cytoplasmic localisation, whereas some cells had both nuclear and cytosolic diffused localisation (Fig. 6D). This implies that while the interaction between FAM83H and CK1 may have some impact on the nuclear localisation of FAM83H, another protein or multiple proteins may have an influence on the nuclear localisation of FAM83H.

### 3.6 Characterisation of FAM83H interaction with CK1 and NCK at the endogenous level

**Fig. 7:**
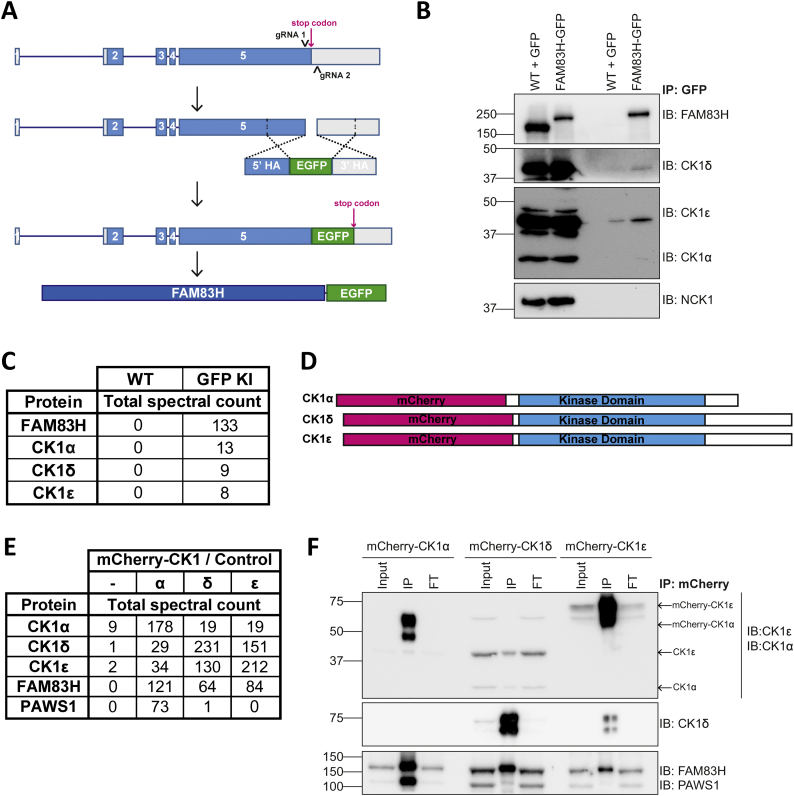
The interaction between FAM83H and CK1 can be detected at an endogenous level. **A** Schematic diagram to represent the mechanism by which A549 *FAM83H*^*GFP/GFP*^ cells were generated using CRISPR/Cas9 genome editing technology. Arrows (^) represent guide RNAs. HA – homology arm. **B** GFP was immunoprecipitated from lysates of A549 *FAM83H*^*WT/WT*^ transiently expressing GFP (WT + GFP) and A549 *FAM83H*^*GFP/GFP*^ (KI) cells. Whole cell lysates (input) and immunoprecipitated proteins (IP) were resolved by SDS-PAGE then analysed by immunoblotting with the antibodies shown. *n* = 1. **C** As **B**, but after resolving samples by SDS-PAGE, the gel was stained in InstantBlue, the gel pieces were excised then processed and proteins digested with trypsin and peptides analysed by mass spectrometry. The table shows total spectral counts of FAM83H, CK1α, CK1δ and CK1ε. n = 1. **D** Schematic representation of mCherry-CK1 knockin proteins. **E** U2OS *CK1*^*mCherry/mCherry*^ cells or control U2OS wild type cells (−) were lysed, resolved by SDS-PAGE, stained in InstantBlue then gel pieces excised then processed and proteins digested with trypsin. Peptides were then analysed by mass spectrometry and total spectral counts are shown in the table. *n* = 1. **F** Cells from **E** were lysed and mCherry immunoprecipitated from cell lysates. Immunoblot shows cell extracts (input), immunoprecipitate (IP) and flow through (FT) samples. *n* = 2.

Thus far, we have primarily employed overexpression systems to investigate FAM83H interactors. Therefore, we sought to validate these interactions at the endogenous level. To do this, we used CRISPR/Cas9 genome editing technology to knockin a gene that encodes enhanced fluorescent GFP (*EGFP*) into the native *FAM83H* gene locus of A549 lung adenocarcinoma cells to encode FAM83H protein with an endogenous C-terminal GFP tag (Fig. 7A). A single cell clone with homozygous C-terminal GFP tag insertion on FAM83H was validated by Western blotting with the disappearance of the endogenous FAM83H signal and concurrent reappearance of a band at a higher molecular weight equivalent to the added GFP (Fig. 7B). Anti-GFP IPs from these cells, but not from WT cells overexpressing GFP control, pulled down FAM83H as well as CK1α, δ and ε (Fig. 7B). Analysis of the anti-GFP IPs by mass fingerprinting also identified FAM83H, CK1α, CK1δ and CK1ε in samples from A549 *FAM83H*^*GFP/GFP*^ cells (Fig. 7C). However, under these conditions, NCK proteins could not be detected in anti-GFP IPs, either by Western blotting or mass fingerprinting (Fig. 5B, C). This suggests the interaction between FAM83H and NCK is potentially transient in nature, or requires a stimulus.

In a similar manner to the generation of A549 *FAM83H*^*GFP/GFP*^ cells, an endogenous *mCherry* tag was knocked into the endogenous *CSNK1A1*, *CSNK1D* and *CSNK1E* gene loci of U2OS cells, which encode CK1α, CK1δ and CK1ε proteins respectively. As such, these cells expressed mCherry-tagged CK1 isoforms (Fig. 7D). mCherry was immunoprecipitated from cell lysates and lysates from U2OS wild type cells were used as a control. IPs were analysed by mass spectrometry. As expected [5], PAWS1 was uniquely identified in mCherry-CK1α IPs by mass spectrometry, but not in mCherry-CK1δ, mCherry-CK1ε nor control IPs (Fig. 7E). FAM83H was identified in mCherry-CK1α, mCherry-CK1δ and mCherry-CK1ε IPs but not control IPs by mass spectrometry (Fig. 7E) and this was validated by Western blotting (Fig. 7F).

### 3.7 Proximity based labelling to investigate the protein components of FAM83H speckles

**Fig. 8:**
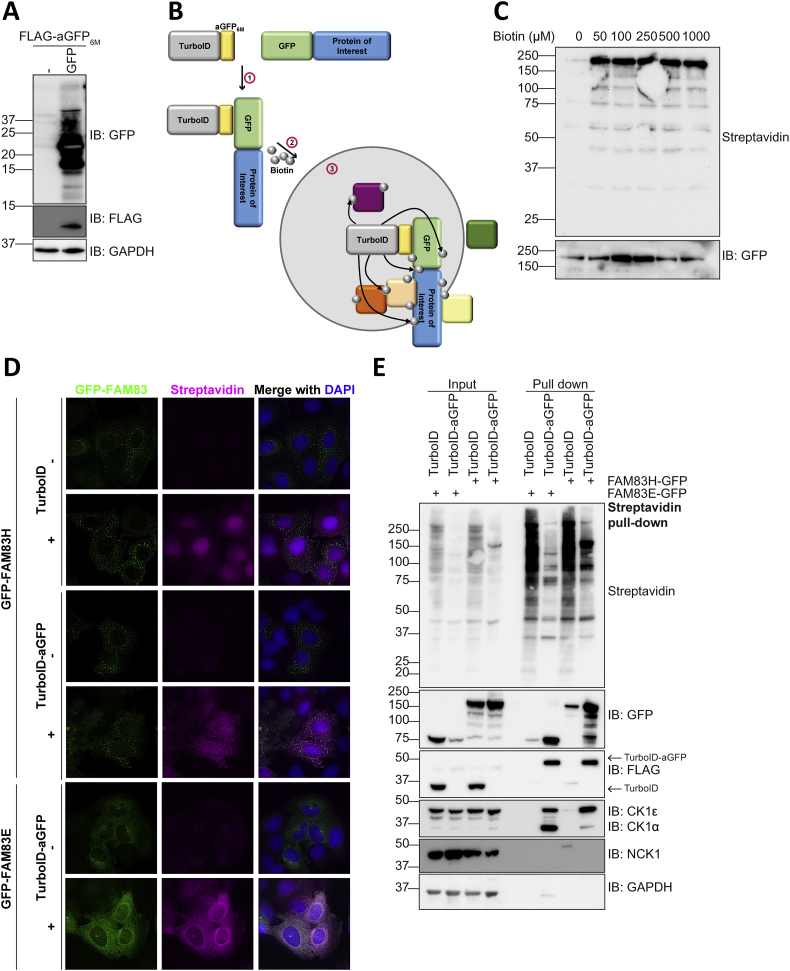
Proximity based labelling to investigate the protein components of FAM83H speckles. **A** U2OS wild type cells were transduced with retroviruses encoding FLAG-aGFP_6M_. These cells were then transfected with a vector encoding GFP or not transfected. Lysates were resolved by SDS-PAGE on a 13% protein gel then immunoblotted (IB) as indicated. *n* = 1. **B** Schematic representation of TurboID-aGFP_6M_ and its mechanism of action. **C** U2OS Flp-In T-REx cells that express FAM83H-GFP under a doxycycline-inducible promoter were infected with retroviruses encoding FLAG-TurboID or FLAG-TurboID-aGFP_6M._ These stable cell lines were induced for 24 h then incubated with the indicated concentrations of biotin for 10 min. After incubation, cells were lysed, resolved by SDS-PAGE and immunoblotted (IB) as indicated. *n* = 1. **D** Cells from **C** were incubated with 50 μM biotin for 10 min, then fixed and stained with streptavidin conjugated to Alexa Fluor 594. DNA was stained with DAPI. Scale bar represents 10 μm. n = 1 **E** U2OS Flp-In T-REx cells expressing FLAG-TurboID or FLAG-TurboID-aGFP_6M_ and expressing either FAM83H-GFP or FAM83E-GFP were incubated with 50 μM biotin for 10 min then lysed and subjected to streptavidin pull down and whole cell lysates (input) and pull downs were immunoblotted (IB) with the antibodies indicated. *n* = 1.

We uncovered that FAM83H disease mutants may cause the mislocalisation of CK1, but do not affect its kinase activity. Moreover, the nuclear localisation of disease mutants is not solely mediated by interaction with CK1. Nevertheless, wildtype FAM83H and disease mutants localise in speckles, while none of the other 7 FAM83 proteins localise to speckles of a similar nature [1,2]. The interaction of FAM83H with CK1 isoforms and NCK does not fully explain the determinants of the speckles. Thus, identifying the key components of these speckles might reveal functional insights into FAM83H. As these speckles appear as compact, condensed structures, we sought to identify the protein components of these speckles by utilising enzymatic proximity-based labelling approaches founded on the high affinity non-covalent interaction between biotin and streptavidin. We utilised TurboID, a mutant derivative of BioID with a substantial improvement in biotinylation kinetics from hours to minutes [35]. TurboID is able to biotinylate proximal proteins upon incubation of cells with biotin for just 10 min [35]. We fused TurboID to an anti-GFP nanobody that contains 6 destabilising mutations (aGFP_6M_) [45], which lead to the degradation of the fusion protein in the absence of GFP (Fig. 8A), thereby reducing the amount of free TurboID-aGFP_6M_. We infected stable U2OS cells that express FAM83H-GFP or FAM83E-GFP upon doxycycline stimulation with retroviruses encoding either FLAG-TurboID-aGFP_6M_ or FLAG-TurboID control. Upon incubation of these cells with biotin, TurboID would be expected to biotinylate proteins proximal to FAM83H-GFP (speckled localisation) and FAM83E-GFP (non-speckled localisation) (Fig. 8B). First, we sought to optimise the concentration of biotin for optimal biotinylation. Western blotting of cell extracts indicated that maximal amount of biotinylated material was detected following incubation of just 50 μM biotin (Fig. 8C), and therefore, for further experiments, cells were incubated in 50 μM biotin.

FLAG-TurboID-aGFP_6M_ or FLAG-TurboID alone control retrovirus transduced cells expressing FAM83H-GFP or FAM83E-GFP were then incubated with 50 μM biotin for 10 min, biotinylation immediately quenched and cells stained with Streptavidin conjugated to Alexa Fluor-594, to recognise biotinylated proteins, and visualised. Proteins biotinylated by TurboID-aGFP_6M_ could be seen to robustly colocalise with FAM83H-GFP or FAM83E-GFP, however proteins biotinylated by FLAG-TurboID alone did not (Fig. 8D). Moreover, when biotinylated proteins were subjected to streptavidin pull-down, FAM83H-GFP and FAM83E-GFP could be robustly detected from streptavidin pull downs and CK1α and CK1ε could also be detected (Fig. 8E).

### 3.8 Iporin and BAG3 may be protein components of FAM83H speckles

**Fig. 9:**
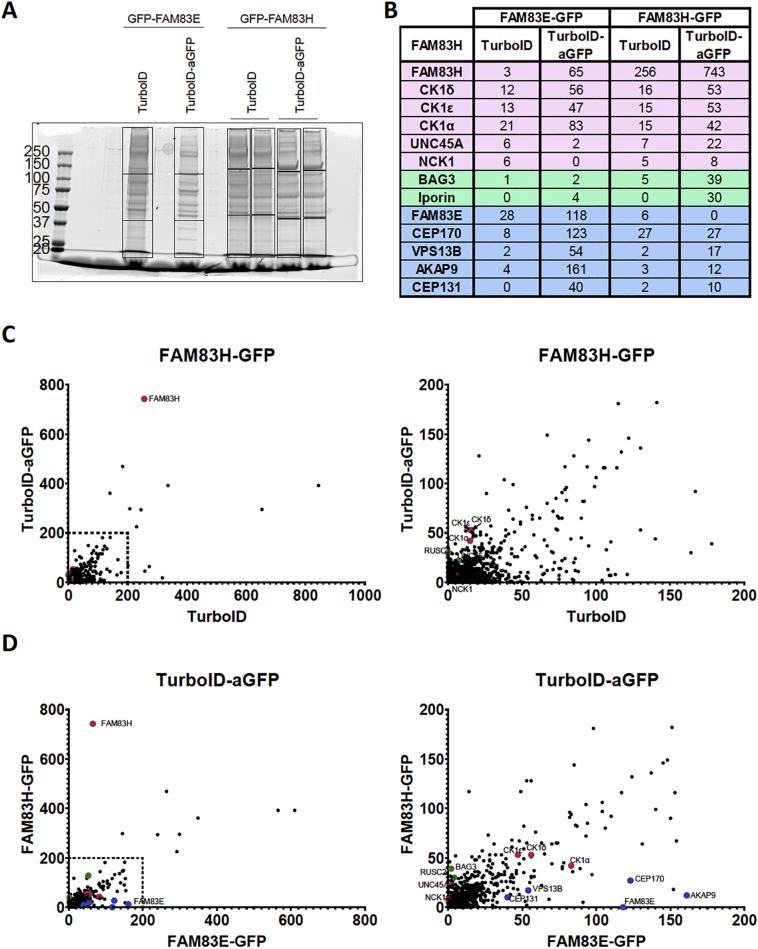
Iporin and BAG3 may be protein components of FAM83H speckles. **A** U2OS Flp-In T-REx cells induced to express FAM83H-GFP or FAM83E-GFP and expressing FLAG-TurboID or FLAG-TurboID-aGFP6M were incubated with 50 μM biotin for 10 min. Cells were then lysed, lysates subjected to Streptavidin pull downs then resolved by SDS-PAGE. Gel pieces were excised as indicated by dotted lines. *n* = 1. **B, C, D** As **A** but after excision, gel pieces were processed then digested with trypsin. Samples were analysed using LC-MS/MS and Scaffold version 4.8.6. *n* = 1. **B** Table showing the total spectral counts of some hits identified via mass spectrometry. n = 1 Proteins in pink indicate known interactors of FAM83H, green indicates novel proximal proteins of FAM83H identified in this paper, blue indicates known interactors of FAM83E. **C, D** Total spectral counts of proteins are plotted where magenta circles indicate a known interactor of FAM83H, green circles indicate a novel hit and blue circles indicate known interactors of FAM83E. n = 1 **C** Scatter plot shows spectral counts from samples in which cells were expressing FAM83H-GFP and FLAG-TurboID or FLAG-TurboID-aGFP_6M_. Left plot shows all proteins identified. Right plot is missing 18 identified proteins and has a smaller scale to enable better visualisation of the identified proteins. *n* = 1. **D** Scatter plot shows spectral counts from samples in which cells were expressing FAM83H-GFP or FAM83E-GFP and FLAG-TurboID-aGFP_6M_. Left plot shows all the proteins that were identified. Right plot is missing 18 identified proteins and has a smaller scale to enable better visualisation of the identified proteins. n = 1.

As TurboID was able to robustly biotinylate known interactors CK1α and CK1ε, this suggested TurboID could be utilised to characterise the components of the FAM83H speckles by TurboID-mediated biotinylation followed by mass spectrometry analysis of the biotinylated proteome. U2OS cells expressing FAM83H-GFP or FAM83E-GFP and FLAG-TurboID or FLAG-TurboID-aGFP_6M_ were incubated with 50 μM biotin for 10 min. Streptavidin pull-downs were employed to enrich biotinylated proteins from extracts. Pull-downs were resolved by SDS-PAGE (Fig. 9A) then the proteins were identified by mass spectrometry (Fig. 9A-D). FAM83H was enriched in cells expressing FAM83H-GFP and TurboID-aGFP_6M_ relative to TurboID control. A similar result was seen from cells expressing FAM83E-GFP, where FAM83E enrichment was seen with TurboID-aGFP_6M_. As expected, CK1α, CK1δ and CK1ε were observed as proximal proteins of both FAM83E and FAM83H (Fig. 9B). Moreover, CEP170, VPS13B, AKAP9 and CEP131, which our previous mass spectrometry data indicates are interactors of FAM83E [1], were also identified as proximal proteins to FAM83E but not FAM83H (Fig. 9B, D). Out of the hundreds of FAM83H proximal proteins identified, two proteins stood out as interesting hits. These include the BAG family molecular chaperone regulator 3 (BAG3) and Interacting protein of rab1 (Iporin) (Fig. 9B-D). Subsequent characterisation of these proteins as potential constituents of the FAM83H-localised speckles needs to be performed.

## 4 Discussion

The cellular and biochemical roles of FAM83H remain poorly characterised. The identification of over 20 unique autosomal dominant *FAM83H* mutations in patients with hypocalcified amelogenesis imperfecta makes it pertinent to understand its function. The FAM83 protein family direct protein kinase CK1 isoforms to distinct subcellular compartments, and FAM83H directs CK1α, δ and ε isoforms to cytoplasmic and nuclear speckle-like structures [1,2]. Here, we show the pathogenic mutants of FAM83H retain association with CK1 isoforms, do not influence the associated CK1 catalytic activity, however localise, and in the process direct CK1 isoforms, to the nucleus. We also report the identification of NCK1/2 as novel interactors of FAM83H in extracts and cells. The SH3–2 and SH3–3 domains of NCKs mediate the interaction with the C-terminal proline-rich domain of FAM83H and therefore this interaction is lost with FAM83H AI truncation mutants. We propose that FAM83H truncation mutants potentially leads to AI pathogenicity through either mis-directed CK1 activity in the nucleus, the loss of FAM83H-NCK association or a combination of both.

We identified a 50 amino acid residue sequence containing two separate proline rich sequences P^915^-V^916^-P^917^-P^918−^V^919^-P^920^ and P^926^-V^927^-P^928^-P^929^-V^930^-P^931^ that, we believe, mediates the interaction between FAM83H and NCK. The proline rich sequences are flanked by positively charged arginine residues N-terminal and C-terminal to the proline rich PVPPVP motifs i.e. E_910_**RR**GSP_915_VPPVP E_921_**RR**SSP_926_VPPVPE_932_**RR**GS. These interaction sites conform to the canonical class I (+xxPxxP) and class II (xPxxPx+) ligands for SH3 binding [46], where + indicates a positively charged residue, which strengthens the interaction with SH3 domains. These two ligands are known to bind SH3 domains in opposite orientations, therefore one may infer that interaction between FAM83H and NCK can occur in two different orientations. Interestingly, DOCK180, like FAM83H, was observed to interact with the SH3–2 and SH3–3 domains of NCK2, where SH3–3 seemed to be the major interaction site [47]. Moreover, NCK may interact with multiple proline rich sequences on DOCK180 [47]. Here, interaction of DOCK180 with two SH3 domains of NCK2, enables NCK2 to have a higher affinity interaction with DOCK180 [47]. Two other proline rich motifs exist outside the identified 50 amino acid stretch, however these motifs and their surrounding residues and charges are vastly different to the aforementioned motifs, and this could therefore explain why these other motifs did not interact with NCK1. We could not robustly detect interaction between FAM83H and NCK at an endogenous level when we performed anti-GFP IPs utilising our A549 *FAM83H*^*GFP/GFP*^ cell line. Although IPs in which we were able to detect interaction between FAM83H and NCK were performed using U2OS and HEK-293 cell lysates, these were under conditions where FAM83H was overexpressed. Moreover, our original proteomics data where FAM83H was overexpressed indicates that the spectral counts of NCK1 and NCK2 in anti-GFP-FAM83H IPs were much lower when compared to CK1 isoforms. As NCK is involved in tyrosine kinase signaling [48], one could speculate that a stimulus may be involved in mediating endogenous interaction between the two proteins. Our initial attempts at identifying a stimulus that induces FAM83H-NCK interaction have been unsuccessful and therefore further investigation into a stimulus, or stimuli, which regulate(s) this interaction at an endogenous level is needed. This may provide key functional insights into how FAM83H mediates intracellular signaling in response to extracellular signals.

The peculiar speckle-like localisation of FAM83H and the truncated mutants, albeit in different subcellular compartments, suggests that understanding the nature and constituents of these speckles could help unravel cellular roles of FAM83H. In our attempts to identify the components of FAM83H speckles, using a modified TurboID proximity labelling we identified some novel proteins, including Iporin and BAG3, as potential constituents of FAM83H-associated speckles. Iporin (interacting protein of Rab1), also known as RUSC2 (RUN and SH3 domain-containing protein 2), is a 161 kDa protein primarily known for its functions in binding Rab1 GTPase [49]. Iporin comprises a single SH3 domain, one RUN domain, two polyproline stretches and one polyglutamic acid stretch. While the function of RUN domains is mostly uncharacterised, RUN domains are believed to have some importance in Rab protein functions. The RUN domain is responsible for the interaction between Iporin and Rab1 and Rab35 GTPases [49,50], where this interaction between Rab1 and Iporin is believed to have some importance in the transport of vesicles [49]. Moreover, Iporin (RUSC2), and RUSC1 (also known as NESCA), are thought to be Adapter Protein complex 4 (AP-4) accessory proteins [51]. AP-4 is an AP complex involved in packaging proteins into vesicles and having roles in the transport and budding of these vesicles. AP-4 packages Autophagy related gene 9A protein (ATG9A) into vesicles, in an Iporin dependent manner and these AP-4 derived vesicles were seen to localise proximal to autophagosomes in cells that were starved of serum [51,52]. Interestingly, BAG3 also has links to the formation of autophagosomes and linking polyubiquitinated protein clients targeted for proteasomal degradation to autophagy [[53], [54], [55]]. BAG3 also links with dynein microtubule motor proteins and 14–3-3 proteins to transport clients to aggresomes [[54], [55], [56]], which are compartments found proximal to the nucleus in which much autophagy occurs [57]. BAG3 is a stress-induced protein which functions as a co-chaperone, interacting with 70 kDa heat shock protein (HSP70) to encourage the release of HSP70 protein clients.

The next steps are to investigate the putative subcellular co-localisation and interactions between FAM83H and Iporin and BAG3 and determine potential biological significance of these interactions. It would also be interesting to investigate whether these two proteins lose interaction with mutants of FAM83H that do not interact with CK1, as the interaction between FAM83H and CK1 is necessary for the speckle-like localisation pattern of FAM83H [9].

Amelogenesis is a complex process, in which the role played by FAM83H is not completely clear. However, we are optimistic that our characterisation of the molecular roles and interactors of FAM83H, will prove to be useful for those conducting research into amelogenesis and will help shed a new light onto novel proteins that may play a role in amelogenesis.

## Funding source

This study was funded by the UK Medical Research Council (MRC) awarded to GPS (grant number MC_UU_12016/3 and MC_UU_00018/6). TTM is supported by the UK MRC DTP studentship and 10.13039/100010269Wellcome Trust Institutional Strategic Support Fund (through School of Life Sciences, 10.13039/100008890University of Dundee).

## Declaration of Competing Interest

None.
